# Genomic variation induced by a low concentration of ethyl methanesulfonate (EMS) in quinoa ‘Longli-4’ variety

**DOI:** 10.1186/s40529-024-00427-x

**Published:** 2024-07-05

**Authors:** Xiaofeng Li, Xiaoyun Cui, Ruilan Ran, Guoxiong Chen, Pengshan Zhao

**Affiliations:** 1 Key Laboratory of Ecological Safety and Sustainable Development in Arid Lands, Northwest Institute of Eco-Environment and Resources, Chinese Academy of Sciences, Lanzhou, 730000 P. R. China; 2 Key Laboratory of Stress Physiology and Ecology in Cold and Arid Regions, Northwest Institute of Eco-Environment and Resources, Chinese Academy of Sciences, Lanzhou, 730000 Gansu Province P. R. China; 3 University of Chinese Academy of Sciences, Beijing, 100049 P. R. China; 4 Academy of Plateau Science and Sustainability, Qinghai Normal University, Xining, 810016 P. R. China

**Keywords:** Quinoa, EMS mutagenesis, Genetic variation, Non-synonymous variation, Stop-gain variation

## Abstract

Quinoa (*Chenopodium quinoa*, 2*n* = 4*x* = 36), a super pseudocereal crop, has been introduced into China nearly 60 years. Many excellent varieties have been developed through massive selection; however, few are developed through mutagenesis breeding. In this study, the ‘Longli-4’ variety, locally cultivated in Gansu province, Northwest China, was selected for experimentation. The grains of ‘Longli-4’ were treated with ethyl methanesulfonate (EMS) at a concentration of 0.8% for 8 h. Nine plants from independent M_2_ families were randomly selected to investigate the mutagenesis effect of EMS on the quinoa genome. The results indicated that the single nucleotide polymorphisms (SNPs) induced by EMS were unevenly distributed across all 18 chromosomes, with an average mutation frequency of 91.2 SNPs/Mb, ranging from 4.5 to 203.5 SNPs/Mb. A significant positive correlation between the number of SNPs and chromosome length was identified through linear model analysis. Transitions from G/C to A/T were the most predominated in all variant categories, accounting for 34.4–67.2% of the mutations, and SNPs were significantly enriched in intergenic regions, representing 69.2–75.1% of the total mutations. This study provides empirical support for the application of low concentration EMS treatment in quinoa breeding.

**Supplementary Information:**

The online version contains supplementary material available at 10.1186/s40529-024-00427-x.

## Introduction

The pseudocereal crop quinoa (*Chenopodium quinoa*), an allotetraploid species (2*n* = 4*x* = 36) (Jarvis et al. 2017), has been consumed for more than 7000 years (Bazile et al. 2013; Gomez-Pando et al. 2019), and the formal breeding of quinoa was initiated in the 1960s (McElhinny et al. 2007). More than 16,000 quinoa accessions have been reported at a global level (Rojas et al. 2013). Normally, these accessions can be categorized into five ecotypes based on the differences in their growth environments, including Sea level, Valley, Altiplano, Salt flat, and Yungas (Gomez-Pando et al. 2019).

Quinoa has been cultivated in China for several decades, leading to the rapid development of diverse varieties through years of selective breeding (Lin et al. 2019). The area devoted to quinoa cultivation has significantly increased from 1,300 hectares in 2014 to 16,600 hectares in 2019, with total yields rising from 2.1 × 10^3^ tons to approximately 28.8 × 10^3^ tons (Cui et al. 2024). More than 20 quinoa varieties have been cultivated crossing over 20 Chinese provinces (regions), each tailed to the unique environment conditions (Cui et al. 2024). The variety known as Longli-4, for instance, is characterized by its white grains, short growth cycle, and high productivity yield, sharing a close relationship with the reference variety PI614886 (Jarvis et al. 2017; Li et al. 2022). Longli-4 has shown adaptability to grow in regions with high-altitudes, arid climates, and a range of cold to cool temperatures (Huang et al. 2020). However, conventional breeding approaches, such as mass- and individual-selection, and cross-breeding typically require several years or even decades to develop a superior variety (Dong et al. 2020). Moreover, ensuring genetic stability across generations is challenging due to quinoa’s high rate of outcrossing (Li et al. 2022). Thus, there is an urgent need to utilize alternative technical methods to breed new quinoa varieties of higher quality.

Ethyl methanesulfonate (EMS) is a mutagenic agent known for its high efficiency, excellent mutagenic effects, relatively simple operation, and low production costs. It has been extensively applied in crop improvement programs for a variety of species, including *Triticum aestivum* (Mishra et al. 2016), *Oryza sativa* (Mohapatra et al. 2014; Shoba et al. 2017), *Zea mays* (Nie et al. 2021), *Brassica napus* (Harloff et al. 2012; Tang et al. 2020), and *Solanum lycopersicum* (Garcia et al. 2016). EMS induces an abundance of point mutations, predominantly causing G to A and C to T transition (Greene et al. 2003; Wang et al. 2023). With the publication of the quinoa genome (Jarvis et al. 2017; Zou et al. 2017; Mangelson et al. 2019), efforts to innovate quinoa germplasm have been gradually gaining momentum. In this context, research related to EMS mutagenesis on quinoa has been conducted. Wang et al. (2021) and Wen et al. (2021) have carried out preliminary investigations into the optimal conditions for EMS mutagenesis on Jingle (Shanxi) and Xinli No.1 quinoa varieties, respectively, determining that 1.5% EMS for 12 h and 1.8% EMS for 3.3 h produced the best results.

An *epidermal bladder cell-free* (*ebcf*) mutant of quinoa was isolated by treating quinoa grains of cultivar 5206 (Titicaca) with 0.2% EMS for 16 h at room temperature (Moog et al. 2022). A previous study found that the REBC gene, encoding a WD40 protein, is involved in the formation of quinoa epidermal bladder cells (EBCs) (Imamura et al. 2020). The *rebc* mutant generated by EMS mutagenesis in the CQ127 variety of quinoa exhibited reduced epidermal bladder cells and a significant decrease in tolerance to abiotic stress (Imamura et al. 2020). In 2018, this group identified two green hypocotyls mutants (*ghy1* and *ghy2*); the candidate genes for both were *CYP76AD1-1* (Imamura et al. 2018). Interestingly, all of these mutants have point mutations that were G to A or C to T transitions, resulting in the formation of non-synonymous substitutions or stop codons (Imamura et al. 2018, 2020; Moog et al. 2022). Cox (2020) constructed a mutant library with 5,030 families by treating the QQ74 variety with EMS, achieving a mutation rate of 21.7 SNPs/Mb. Subsequently, Parker (2022) performed a preliminary analysis of the candidate genes for some of these mutant families with a reduced height phenotype. In one family, the candidate gene (*GAI1*) related to the short phenotype was identified, which formed missense or stop-gained mutations caused by G/C to A/T transitions (Parker 2022). In these studies, G/C to A/T transitions were the primary types of point mutations induced by EMS, and the variant positions were predominantly located in the intergenic regions (Cox 2020; Parker 2022). This phenomenon was also observed by Mestanza et al. (2019) with one mutation per 203 Kb. Although EMS mutagenesis of quinoa was conducted, the characteristics of SNPs generated by lower concentrations of EMS on each chromosome and the relationship between the number of SNPs and the chromosome length were relatively under reported in previous studies.

Longli-4, a widely cultivated quinoa variety in China with beneficial traits developed through years of breeding (Huang et al. 2020), has a genomic makeup closely related to the reference variety PI614886 (Li et al. 2022). This relatedness minimizes genomic background variation, facilitating the detection of the SNPs induced by EMS treatment. In this study, a lower concentration of EMS (0.8%) was utilized to create a mutant library for Longli-4. Nine mutants, exhibiting no significant phenotypic variations, were selected for further analysis. Whole-genome resequencing was conducted to explore the characteristics of the EMS-induced SNPs. We also characterized the chromosomal distribution patterns of SNPs with non-synonymous and stop-gain mutations. Our findings lay the groundwork for future mutagenesis breeding strategies and the construction of comprehensive mutant libraries in quinoa.

## Materials and methods

### Plant materials and genome resequencing

Approximately 400 g of Longli-4 variety grains (M_0_) were treated with 0.8% EMS for 8 h according to protocol provided by the Plant Seed EMS mutagenesis kit (GenMED Scientifics Inc. U.S.A). The resulting M_1_ seeds were then sown in the experimental field in Haiyuan, Ningxia province, in 2019, employed the method delineated by Li et al. (2021). Upon reaching full maturity, M_1_ plants were randomly selected, from which 100 individual plants were used to harvest M_2_ seeds separately, while the seeds from the remaining plants were collected in bulk. In May 2020, approximately M_2_ 15–20 seeds from each of the 100 M_1_ individuals were sown in pots (20 cm × 30 cm), which were filled with nutrient-rich soil. At the same time, seeds of the unmutated Longli-4 variety were sown as the control specimens. Two months post-planting, young leaves were sampled from each plant for sequencing purposes. The mutant plants were labeled R2-R10, corresponding to individual plants #102, #118, #120, #143, #15, #20, #69, #71, and #84, while five Longli-4 control plants were labeled R11-R15. These samples were flash-frozen in liquid nitrogen and then sent to Biomarker Technologies (Beijing, China) for genomic analyses. Sequencing libraries were constructed following the standard Illumina protocol, and paired-end sequencing was performed on the Illumina platform. It is important note that an additional CA3-1 variety, initially labeled as R1, was also included in the sequencing process. However, data from R1 were deleted from the analysis presented in this study, with the focus being solely on the 14 remaining individuals (R2-R15).

### SNP calling and statistical analyses

Based on the MEM algorithm of Burrows-Wheeler Aligner (Li and Durbin 2009) (BWA 0.7.10-r789), the clean reads obtained after filtering were aligned back to the reference genome (PI614886) (Jarvis et al. 2017). A total of 200.7 Gb clean reads were acquired, with a Q30 value of 93.5%. The average mapping rate was 99.4%, of which the properly mapped rate was 96.6%, and the average sequencing depth was 9X. Redundant reads were filtered by Picard (available at http://sourceforge.net/projects/picard/), followed by single nucleotide polymorphism (SNP) calling using the Genome Analysis Toolkit (GATK version 3.8) with the default setting (McKenna et al. 2010).

To characterize the variation of each mutant, the SNPs were further filtered as follows: (1) to ensure the genetic background consistency of the wild-type (WT) control plants, SNPs caused by individual differences were excluded to obtain SNPs shared among the five WTs; (2) to account for the genotype difference between the Longli-4 WT and the reference genomic line (PI614886), SNP variations resulting from genotype divergence were deleted to obtain the SNPs common to the WT and the reference genome; (3) to identify SNPs generated by EMS mutagenesis, SNPs shared among the nine mutants were removed; (4) this study mainly focuses on chromosomal variants; thus SNPs that could not be mounted into the 18 chromosomes were discarded; (5) it was hypothesized that each mutation occurred independently, so the loci involved in the variation were specific to each individual, and therefore the SNPs obtained were unique to at least one of the nine mutants, such as R2=(A, A), R3 = R4=…=R10=(C, C); or R2=(A, A), R3=(A, C), R4 = R5=…=R10. The above steps ensure that the screened SNPs were generated by EMS, forming the basis for subsequent analyses.

For each mutant, a comparative analysis with WT was performed separately using RStudio (version R4.3.0) to remove the SNPs shared by mutants and WT, as well as those identified as “N”. The total number of SNPs and those identified as non-synonymous mutations were characterized for their distribution on each chromosome using the *CMplot* R package (Yin 2022). The number of total, homozygous, non-synonymous, and homozygous and non-synonymous mutations was calculated for each mutant using *do* (Zhang and Jin 2021) and *data.table* (Dowle and Srinivasan 2021) R packages to investigate the distribution frequency of SNPs induced by EMS on the chromosomes. The base variation pattern of the SNPs was analyzed to confirm if the EMS-induced mutations exhibited bias. The number and relative abundance of SNPs were statistically measured based on variation types and genomic positions using *do* (Zhang and Jin 2021) and *data.table* (Dowle and Srinivasan 2021) R packages. Stacked bar charts were drawn to visualize these statistics. Furthermore, the relationship between total SNPs, non-synonymous mutations, stop-gain variations, and chromosome length was evaluated utilizing linear models. The dendrograms of hierarchical clustering were generated using *ggplot2* R package (Wickham 2016) and *tanglegram* function of *dendextend* R package (Galili 2015). Additionally, GO enrichment analysis was performed for genes containing non-synonymous mutations and stop-gain variants of the SNPs using *clusterProfiler* R package (Wu et al. 2021), with the aid of annotated information from SnpEff (Cingolani et al. 2012).

## Results

### The variation characteristics of SNPs in nine mutants

**Fig. 1:**
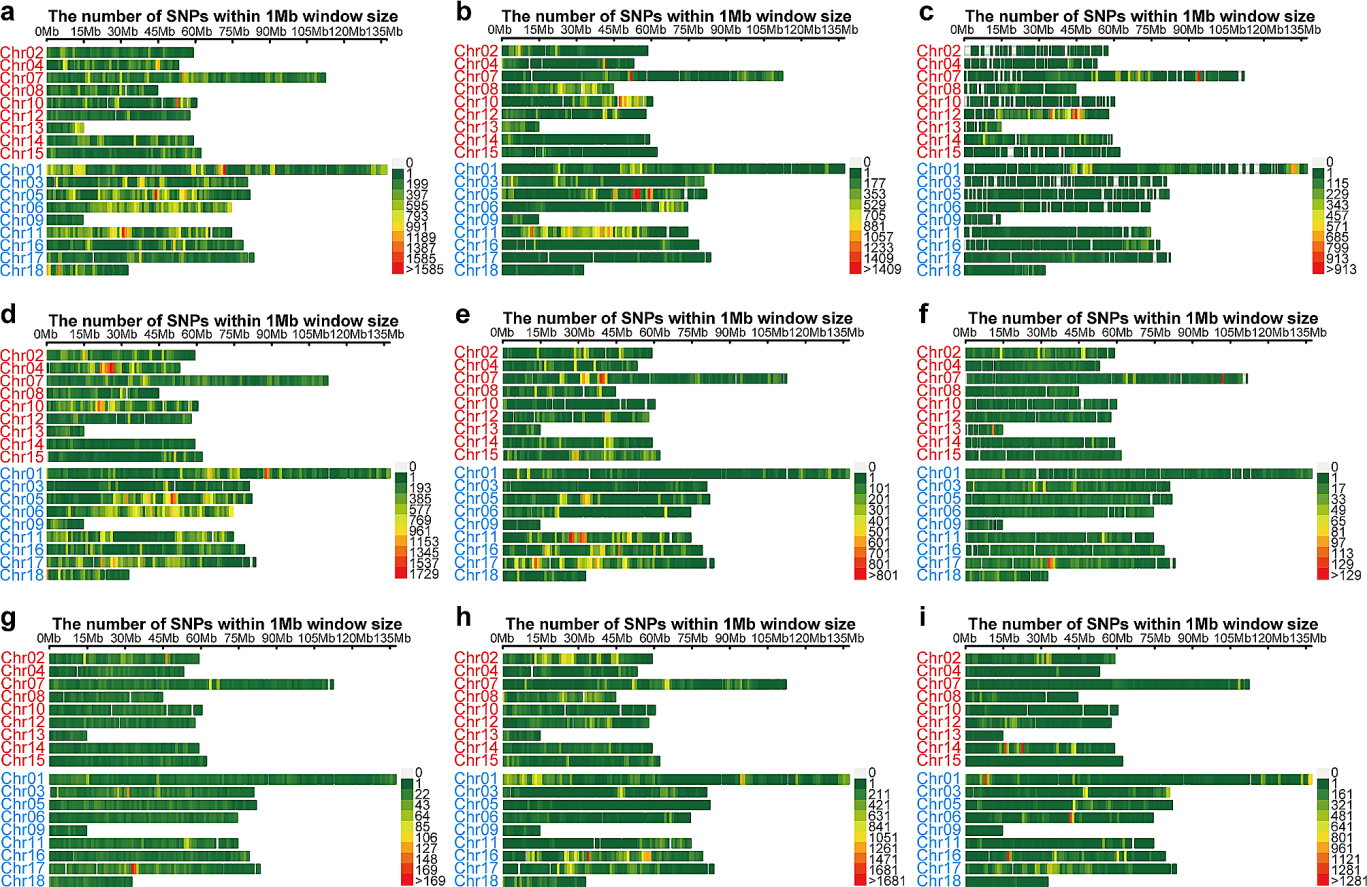
The distribution patterns of SNPs on 18 chromosomes in nine mutants (R2-R10) in comparison with the Longli-4 wild type plants (**a-i**). The chromosomes of the A sub-genome are shown in red, and those of the B sub-genome are shown in pale blue

**Fig. 2:**
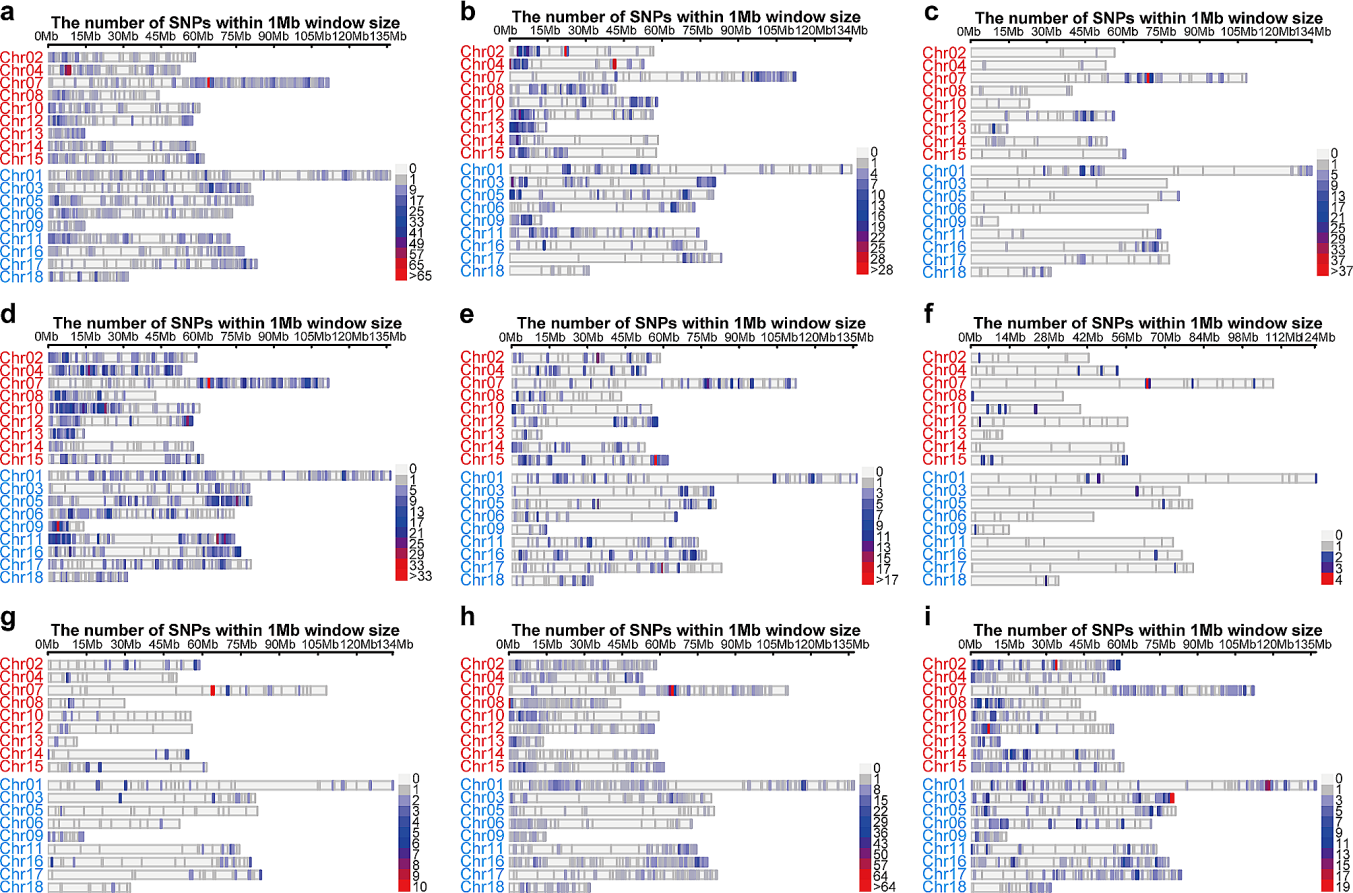
The distribution patterns of non-synonymous variations on 18 chromosomes in nine mutants (R2-R10) in comparison with the Longli-4 control plants (**a-i**). The chromosomes of the A sub-genome are shown in red, and those of theB sub-genome are shown in pale blue

The analysis of nine mutants and five Longli-4 individuals yielded a total of 4,370,603 SNPs for variant calling. After initial filtering, 1,194,380 SNPs were retained for in-depth analysis. Individual screening of each mutant revealed varying SNP counts, with R2-R10 exhibiting 250,965, 129,264, 39,608, 236,557, 84,668, 10,908, 18,282, 151,720, and 78,582, respectively. The distribution of SNPs was visualized on chromosome maps (Figs. 1 and 2). These SNPs were not uniformly dispersed across the chromosomes, with distinct hotspots-areas of high SNP concentration-emerging among different mutants (Figs. 1 and 2). For example, R2 showed notable hotspots on Chromosomes 01, 05, 10, and 11 (Fig. 1a); R3 on Chromosomes 05 and 07 (Fig. 1b); R4 on Chromosomes 07 and 12 (Fig. 1c); R5 on Chromosomes 01, 04, and 05 (Fig. 1d); R6 on Chromosomes 07 and 11 (Fig. 1e); R7 on Chromosome 07 (Fig. 1f); R8 on Chromosome 17 (Fig. 1g); R9 on Chromosome 16 (Fig. 1h); and R10 on Chromosomes 01, 06, 14, and 16 (Fig. 1i).

When comparing non-synonymous SNP variations, which can lead to changes in amino acid sequences and potentially alter protein function, a marked decrease numbers was noted, as well as distinct hotspot patterns (Fig. 2). For instance, hotspots for non-synonymous SNPs in R2 were found on chromosomes 04 and 07 (Fig. 2a), for R3 on Chromosomes 02 and 04 (Fig. 2b), for R4 on Chromosome 07 (Fig. 2c), for R5 on Chromosomes 07 and 09 (Fig. 2d), for R6 on Chromosome 15 (Fig. 2e), and for both R7 and R8 on Chromosome 07 (Fig. 2f and g), for R9 on Chromosomes 07 and 08 (Fig. 2h), and for R10 on Chromosomes 02, 12, and 03 (Fig. 2i).

**Fig. 3:**
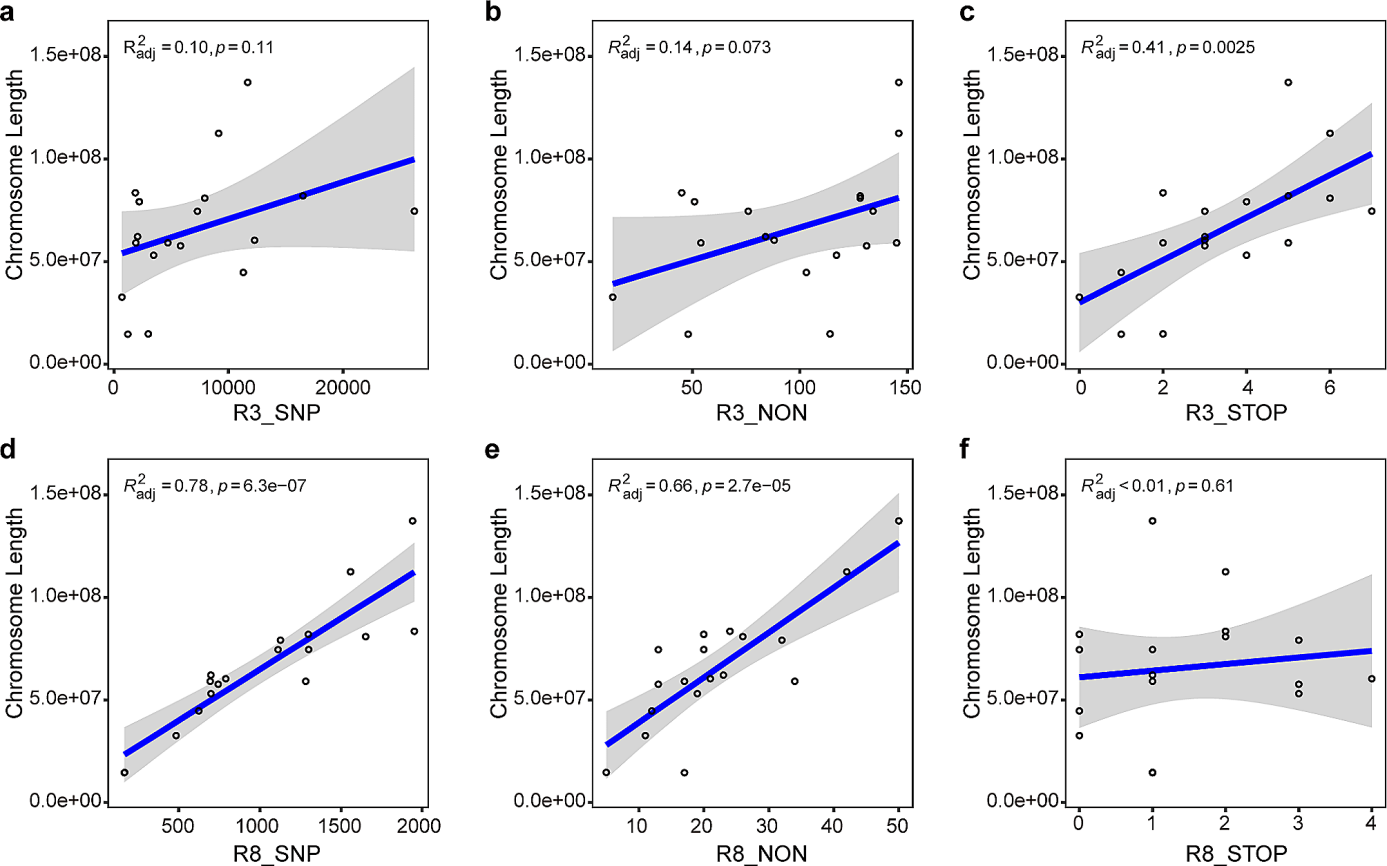
Linear regression analyses of the chromosome length and SNPs number in R3 and R8. Linear regression diagram of the chromosome length against total SNPs, non-synonymous, and stop-gain mutations for R3 (**a-c**) and R8 (**d-e**)

**Fig. 4:**
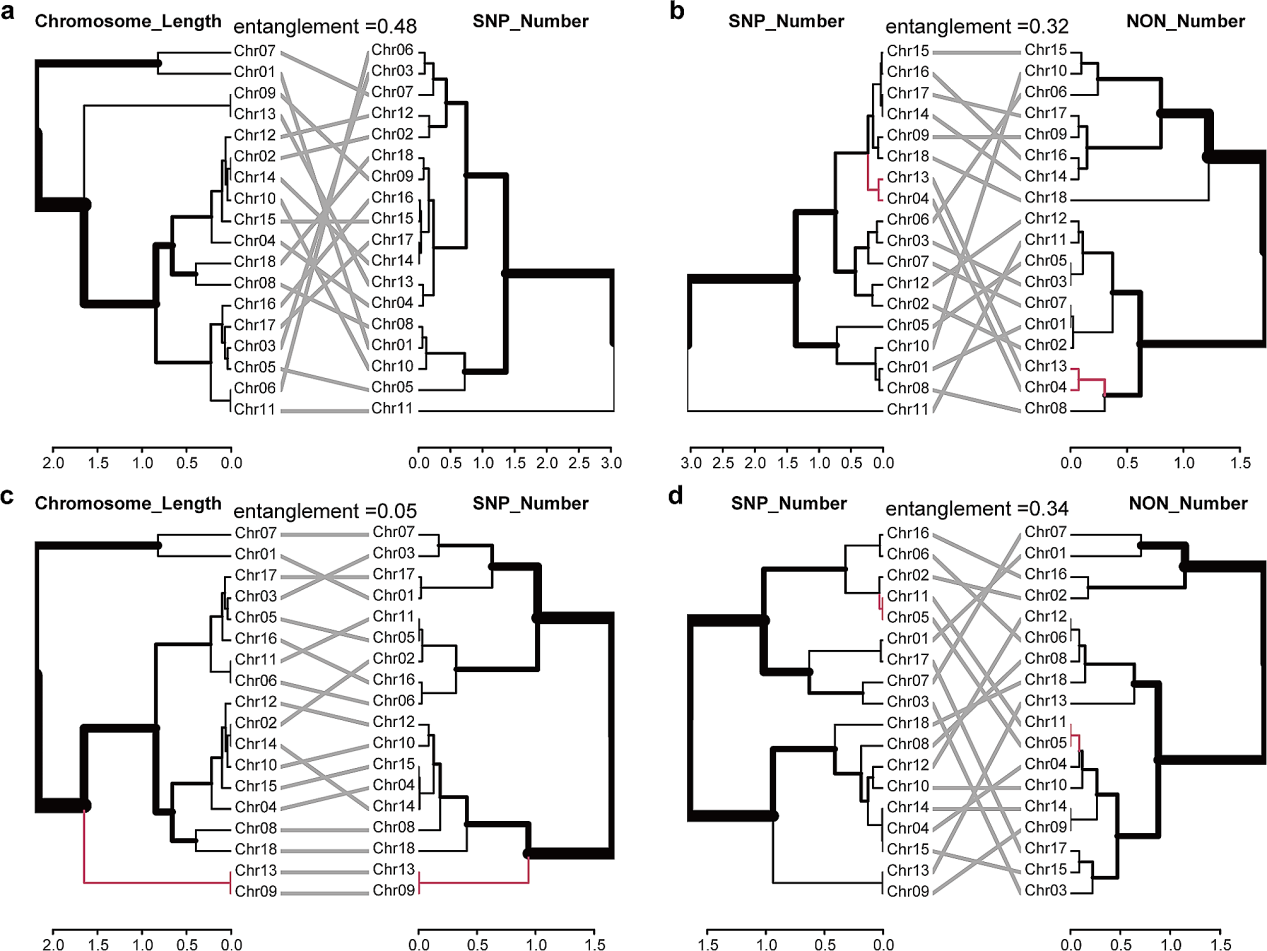
Dendrogram topology comparison of chromosomes length and total SNPs number, and comparison of total SNPs and non-synonymous mutations for R3 (**a, b**) and R8 (**c, d**). The connection lines are in red to highlight two sub-trees that are present in both dendrograms

To estimate the mutagenesis effects of EMS at a low concentration (0.8%), we analyzed the frequency of the SNP occurrence individually for each mutant. Table 1 showed that the SNP frequencies vary significantly among mutants and also among different chromosomes within the same mutant. For the nine mutants examined, the lowest SNP frequency was found in mutant R7, with an average of 4.5 SNPs/Mb (range from 1.9 to 9.8 SNPs/Mb), while the highest was in mutant R2, with 203.5 SNP/Mb (range from 65.3 to 366.6 SNPs/Mb) (Table 1). Linear models and dendrograms topology comparison were constructed to explore potential relationships between the total number of SNPs, non-synonymous mutations, stop-gain mutations, and chromosome lengths for each mutant (as shown in Figs. 3 and 4, and Fig. S1-S9). Strong correlations between the total numbers of SNPs and the chromosome lengths were observed in eight out of nine mutants, with *R*^*2*^ ranging from 0.20 to 0.78 (*p* values ranging from 6.3e-07 to 0.035; Fig. 3d; Fig. S1-S9). The strongest correlation was identified in mutant R8 (*R*^*2*^ = 0.78, *p* = 6.3e-07; Fig. 3d), while no significant correlation was detected in mutant R3 (*R*^*2*^ = 0.10, *p* = 0.11; Fig. 3a). The dendrogram topology comparison showed a similar pattern to the correlation analyses, with entanglement values for mutants R2 and R4-R10 ranging from 0.05 to 0.42. However, mutant R3 had a higher entanglement value of 0.48 (Fig. 4a and c; Fig. S1 and S3-S9). Furthermore, strong correlations between the number of non-synonymous mutations and chromosome length were detected in all mutants except R3, with *p*-values ranging from 1.7e-05 to 0.018 (Fig. 3b and e; Fig. S1-S9). As for stop-gain variants’ correlation with chromosome length, strong correlations were found only in mutants R2, R3, R4, R5, and R9, with *p*-values ranging from 2.7e-05 to 0.024 (Fig. 3c and f; Fig. S1-S9).

**Table 1:** The SNP mutation frequency on each chromosome (SNP/Mb)

	R2	R3	R4	R5	R6	R7	R8	R9	R10	Average
Chr02	138.0	79.6	3.3	189.1	66.6	8.7	21.7	285.4	93.2	98.4
Chr04	218.0	65.1	11.5	366.7	65.4	2.7	13.1	73.3	28.3	93.8
Chr07	210.5	81.3	59.8	164.2	82.6	3.6	13.9	103.4	33.3	83.6
Chr08	156.8	252.8	33.8	125.0	66.0	3.9	14.0	244.8	53.8	105.7
Chr10	172.9	203.3	5.0	316.1	16.5	2.6	13.1	71.9	23.6	91.7
Chr12	110.0	100.8	168.9	95.8	62.4	3.4	12.9	139.5	70.0	84.9
Chr13	268.8	203.4	50.7	142.2	45.5	9.6	11.4	115.1	25.6	96.9
Chr14	169.4	32.3	64.7	71.9	67.6	2.8	11.7	109.4	140.1	74.4
Chr15	113.3	33.1	6.5	88.3	109.8	1.9	11.2	98.4	22.3	53.9
Chr01	238.4	85.1	54.6	172.3	40.6	3.1	14.1	164.2	68.5	93.4
Chr03	205.3	97.9	5.7	74.6	24.6	7.3	20.4	81.2	70.1	65.2
Chr05	313.8	201.3	9.9	309.1	69.3	3.1	15.8	21.3	63.4	111.9
Chr06	366.6	97.7	8.4	396.0	39.9	3.4	14.9	63.0	76.2	118.5
Chr09	65.3	83.2	11.7	171.7	13.9	4.7	11.3	66.9	21.9	50.1
Chr11	363.8	351.9	15.1	244.2	140.9	4.1	17.4	70.9	38.8	138.6
Chr16	149.8	27.9	22.1	129.6	104.0	2.5	14.2	304.8	118.2	97.0
Chr17	142.7	22.4	28.0	275.8	146.7	9.8	23.4	138.2	126.4	101.5
Chr18	260.2	21.3	23.5	209.8	46.4	4.3	14.8	129.8	25.0	81.7
Sum	3663.6	2040.4	583.2	3542.4	1208.7	81.5	269.3	2281.5	1098.7	3663.6
Average	203.5	113.4	32.4	196.8	67.1	4.5	15.0	126.7	61.0	91.2

**Fig. 5:**
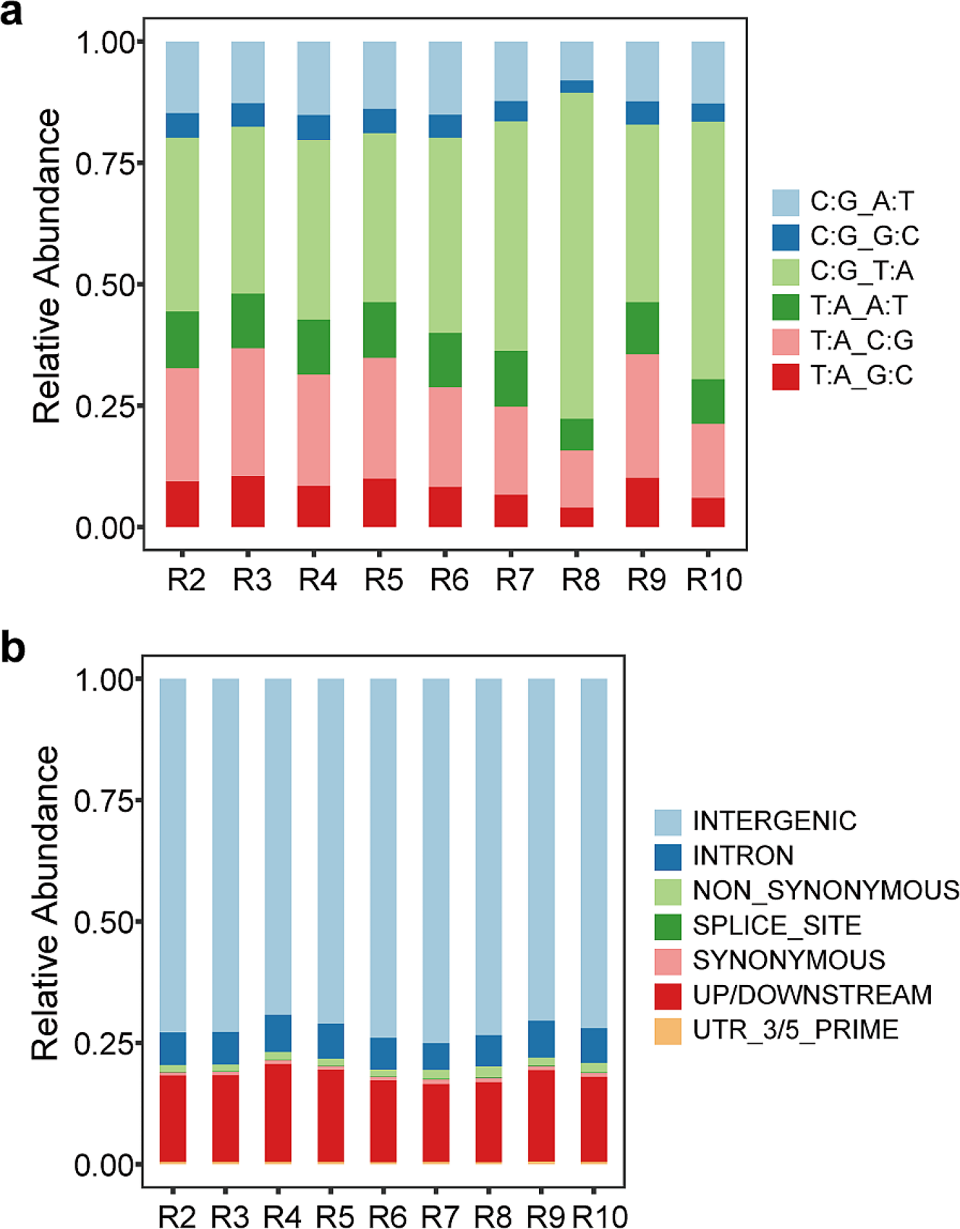
Stacked bar charts of the total variations in R2-R10 individuals. (**a**) SNPs are categorized into six groups based on transitions and transversions, and the percentage of each group in R2-R10 mutants is presented as a stacked bar. (**b**) SNPs are categorized into seven groups based on annotation, and relative abundance values of seven groups in R2-R10 are shown as a stacked bar

Typically, SNPs are categorized as either transitions (purine-to-purine [G/A] or pyrimidine-to-pyrimidine transitions [C/T]) or transversions. Transitions are more common than transversions. To assess EMS-induced SNP preferences in quinoa, we independently analyzed the base variation of SNPs for each mutant and chromosome. Most SNPs were transitions, accounting for 59.1%, 60.7%, 60.0%, 59.7%, 60.8%, 65.4%, 78.9%, 62.0%, and 68.3% of the total variations across the nine mutants, respectively, a proportion significantly higher than that of the transversions (Fig. 5a; Fig. S10). Within the transitions, the frequency of C/G to T/A was higher than that of T/A to C/G in all nine mutants (ranging from 34.4 to 67.2% vs. 11.7 to 26.3%). For transversions, the relative percentages of C/G to A/T, T/A to A/T, and T/A to G/C were quite similar across the board, whereas the C/G to G/C transformation represented the lowest proportion of total SNPs in all mutants (Fig. 5a; Fig. S10). According to the SNP annotations using snpEFF, most SNPs were located in the intergenic regions (ranging from 69.2 to 75.1%), while 16.0–20.2% SNPs were found in upstream and downstream regions (Fig. 5b; Fig. S11). Detailed analysis revealed that only 1.3–2.2% SNPs resulted in non-synonymous mutations across the nine mutants (Fig. 5b; Fig. S11).

### GO enrichment of the non-synonymous and stop-gain SNPs

**Fig. 6:**
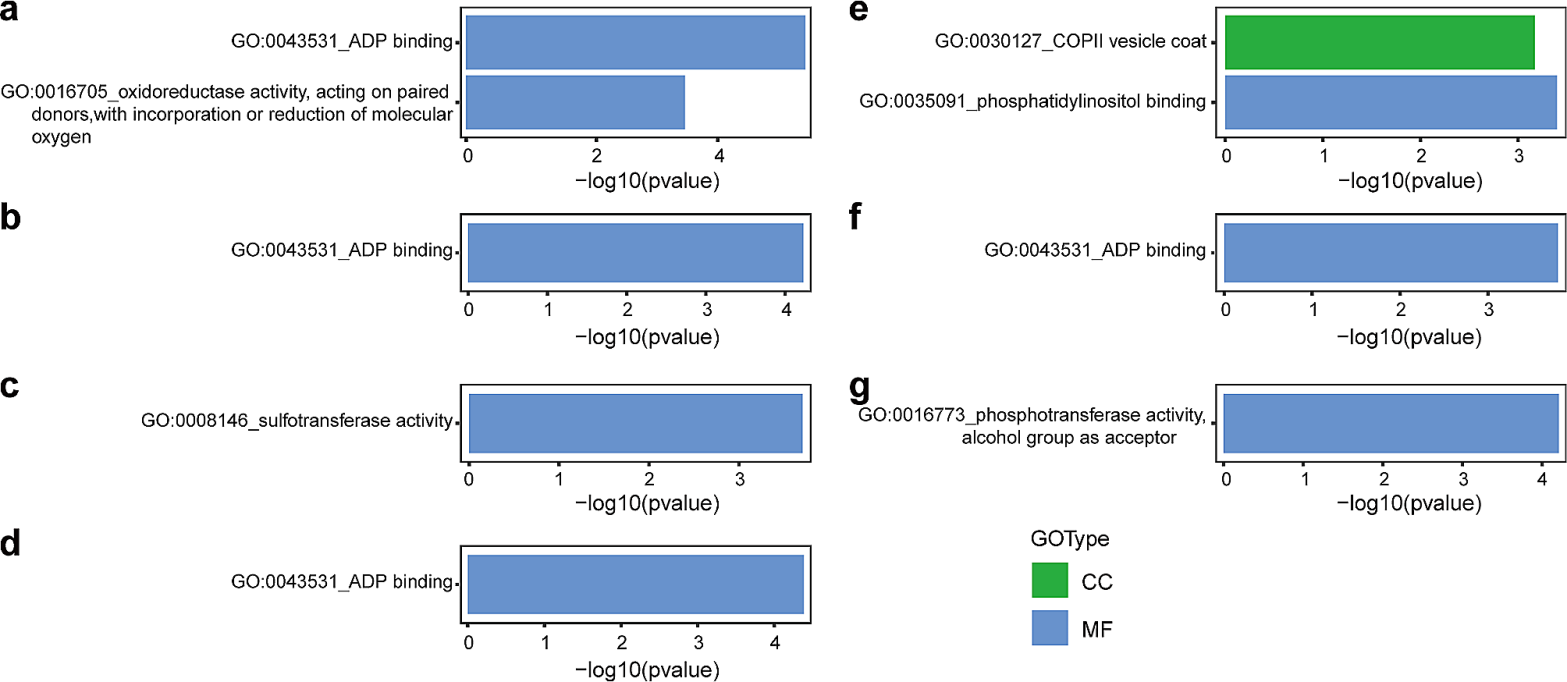
GO enrichment of the genes with non-synonymous SNPs for R2-R5 (**a-d**) and R8-R10 (**e-g**) individuals

Non-synonymous mutations and the introduction of premature stop-codon typically result in phenotypic variation. The number of genes affected by non-synonymous mutations and stop-gain SNPs was determined for each mutant (refer to Tables 2 and 3). The average number of genes with non-synonymous mutations spanned from 10.6 to 144.4 across 18 chromosomes in the nine mutants (Table 2), while the number of genes with the stop-gain SNPs was notably lower, ranging from 1.0 to 6.3 (Table 3). These stop-gain SNPs accounted for only 0.03–0.09% of total variation observed in all mutants. Gene Ontology (GO) enrichment analysis was employed to identify the potential functions of the genes affected by these mutations. For the R2 mutant, two GO terms were significantly enriched: ‘ADP binding’ (GO:0043531, *p* = 2.48e-06) and ‘oxidoreductase activity, acting on paired donors, with incorporation or reduction of molecular oxygen’ (GO:0016705, *p* = 0.0002 (Fig. 6a). The mutants R3, R4, R5, R9, and R10 each had a single GO term significantly enriched, specifically ‘ADP binding’ (GO:0043531, *p-*values ranging from4.11e-05 to 5.88e-05), ‘sulfotransferase activity’ (GO:0008146, *p* = 0.0002), and ‘phosphotransferase activity, alcohol group as acceptor’ (GO:0016773, *p* = 6.21e-05) (Fig. 6b-d, f and g). For the R8 mutant, two GO terms were enriched: ‘phosphatidylinositol binding’ (GO:0035091, *p* = 0.0004), and ‘COPII vesicle coat’ (GO:0030127, *p* = 0.0007 (Fig. 6e). No GO terms were significantly enriched in the R6 and R7 mutants.

**Table 2:** The number of genes with non-synonymous SNPs

	R2	R3	R4	R5	R6	R7	R8	R9	R10
Chr02	121	116	4	133	55	7	30	133	110
Chr04	156	91	7	227	56	16	18	102	51
Chr07	330	129	122	282	110	25	34	207	110
Chr08	88	85	15	84	40	3	12	130	70
Chr10	109	68	4	248	33	12	21	124	45
Chr12	151	99	62	117	56	14	13	87	72
Chr13	69	89	22	60	13	6	5	55	22
Chr14	121	41	33	45	43	7	16	63	70
Chr15	132	73	11	91	95	12	23	136	51
Chr01	248	119	83	213	91	21	48	266	145
Chr03	156	90	5	67	36	12	23	98	107
Chr05	161	97	15	218	57	11	19	32	78
Chr06	137	62	6	148	33	7	13	72	65
Chr09	34	42	4	79	10	6	17	33	24
Chr11	207	104	17	245	59	7	19	102	52
Chr16	135	42	60	135	84	6	32	169	104
Chr17	130	42	23	131	66	12	24	90	90
Chr18	73	12	23	76	28	7	11	55	28
Sum	2558	1401	516	2599	965	191	378	1954	1294
Average	142.1	77.8	28.7	144.4	53.6	10.6	21.0	108.6	71.9

**Table 3:** The number of genes with stop-gain SNPs

	R2	R3	R4	R5	R6	R7	R8	R9	R10
Chr02	6	5	1	4	2	0	1	8	7
Chr04	7	4	0	11	5	3	3	5	3
Chr07	11	6	4	12	3	2	2	10	4
Chr08	3	1	0	5	1	0	0	4	4
Chr10	3	3	0	11	0	1	4	2	1
Chr12	8	3	0	3	5	0	3	4	7
Chr13	3	2	1	2	0	0	1	0	1
Chr14	2	2	1	0	3	1	1	2	4
Chr15	6	3	1	2	2	2	1	3	5
Chr01	8	6	4	11	3	1	1	15	6
Chr03	9	6	1	3	0	0	2	5	3
Chr05	9	5	1	6	3	2	0	3	5
Chr06	7	3	0	16	1	1	0	3	1
Chr09	2	1	1	2	2	0	1	0	2
Chr11	13	7	0	4	3	0	1	3	3
Chr16	7	4	2	5	5	1	3	4	4
Chr17	3	2	1	6	5	0	2	5	11
Chr18	6	0	0	2	1	1	0	2	1
Sum	113	63	18	105	44	15	26	78	72
Average	6.3	3.5	1.0	5.8	2.4	0.8	1.4	4.3	4.0

**Fig. 7:**
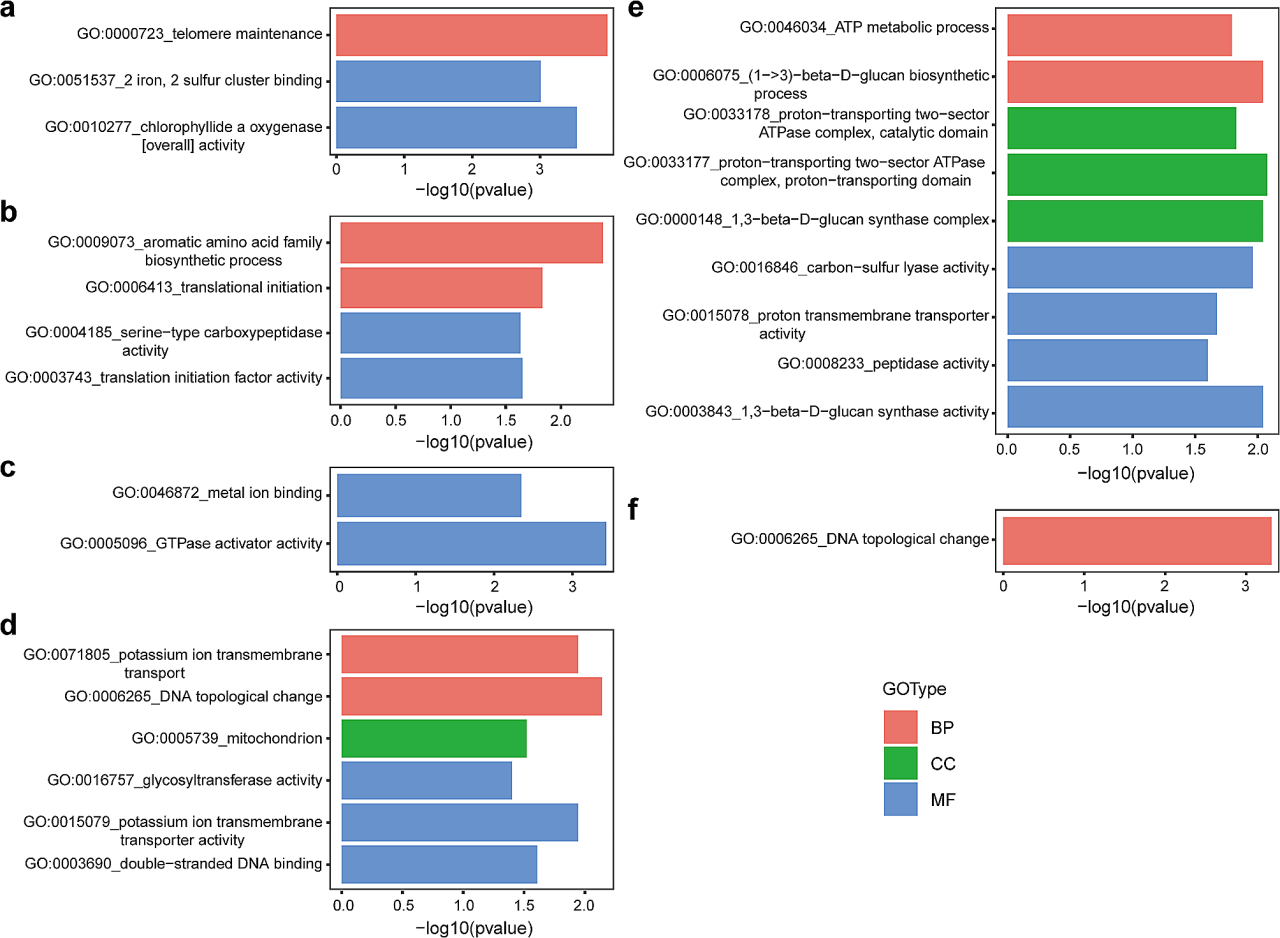
GO enrichment of the genes with stop-gain SNPs for R2 (**a**), R4 (**b**), R6-R9 (**c-f**) individuals

Consistent with non-synonymous mutations, significant enrichment of GO terms was detected in individuals R2, R4, R8, and R9 with stop-gain mutations. For example, GO terms related to ‘telomere maintenance’ (GO:0000723, *p* = 0.0001), ‘chlorophyllide a oxygenase [overall] activity’ (GO:0010277, *p* = 0.0003), and ‘2 iron, 2 sulfur cluster binding’ (GO:0051537, *p* = 0.0010) were notably present in R2 (Fig. 7a). In R4, enriched GO terms included ‘aromatic amino acid family biosynthetic process’ (GO:0009073, *p* = 0.0041), ‘translational initiation’ (GO:0006413, *p* = 0.0148), ‘translation initiation factor activity’ (GO:0003743, *p* = 0.0223), and ‘serine-type carboxypeptidase activity’ (GO:0004185, *p* = 0.0233) (Fig. 7b). For R8, eight GO terms were identified, such as ‘ATP metabolic process’ (GO:0046034, *p* = 0.0161), ‘(1->3)-beta-D-glucan biosynthetic process’ (GO:0006075, *p* = 0.0090), ‘1,3-beta-D-glucan synthase complex’ (GO:0000148, *p* = 0.0090), ‘proton-transporting two-sector ATPase complex, proton-transporting domain’ (GO:0033177, *p* = 0.0084), ‘proton-transporting two-sector ATPase complex, catalytic domain’ (GO:0033178, *p* = 0.0148), ‘1,3-beta-D-glucan synthase activity’ (GO:0003843, *p* = 0.0090), ‘peptidase activity’ (GO:0008233, *p* = 0.0250), ‘proton transmembrane transporter activity’ (GO:0015078, *p* = 0.0212), and ‘carbon-sulfur lyase activity’(GO:0016846, *p* = 0.0110) (Fig. 7e). For R9, the GO term ‘DNA topological change’ (GO:0006265, *p* = 0.0005) was highlighted (Fig. 7f). In contrast to non-synonymous mutations, enriched GO terms were detected for genes with stop-gain SNPs in R6 and R7, but not in R3 and R5. In R6, ‘GTPase activator activity’ (GO:0005096, *p* = 0.0004) and ‘metal ion binding’ (GO:0046872, *p* = 0.0045) were significantly enriched (Fig. 7c). Meanwhile, in R7, six GO terms such as ‘DNA topological change’ (GO:0006265, *p* = 0.0072), ‘potassium ion transmembrane transport’ (GO:0071805, *p* = 0.0114), ‘mitochondrion’ (GO:0005739, *p* = 0.0300), ‘double-stranded DNA binding’ (GO:0003690, *p* = 0.0246), ‘potassium ion transmembrane transporter activity’ (GO:0015079, *p* = 0.0114), and ‘glycosyltransferase activity’ (GO:0016757, *p* = 0.0396), were significantly enriched (Fig. 7d).

## Discussion

EMS mutagenesis efficiency is influenced by the concentration of the mutagen, the duration of treatment, and seed size, therefore optimal conditions for mutagenesis vary among different varieties (Chen et al., 2023). Previous studies have identified the most suitable concentrations and times for EMS mutagenesis in various quinoa varieties: 1.5% for 12 h in Jingle (Wang et al. 2021), 1.8% for 3.3 h in Xin NO.1 (Wen et al. 2021), 2.0% or 2.5% for 6 h in QQ74 (Cox 2020), and 2.0% for 8 h in Regalona-Baer (Mestanza et al. 2019). While these higher concentrations are advantageous for constructing mutant libraries, they may not be ideal for quinoa breeding due to the resulting severe phenotypic variation (Cox 2020). In comparison to high doses mutagenesis, lower-dose may reduce the number of deleterious mutations and decrease the toxicity to the seeds and/or seedlings, thereby increasing the potential for successful mutations and improving the survival rate, as well as possibly enhancing the resistance of the plant to adverse environments (Hu et al. 2017; Shen 2018; Wang 2018; Gillmor and Lukowitz 2020; Yan 2023). In the current study, Longli-4 variety seeds were treated with a significantly lower concentration of EMS at 0.8%, which did not cause obvious phenotypic variation in M_1_ and M_2_ plants. However, the average mutation rate was 91.2 SNPs/Mb (ranging from 4.5 to 203.5 SNPs/Mb; Table 1), surpassing the rates observed in QQ74 (21.8 SNPs/Mb) (Cox 2020) and Regalona-Baer (4.93 SNPs/Mb) (Mestanza et al. 2019) varieties of quinoa. These findings suggest that lower EMS concentrations can induce substantial genetic variation while inflicting minimal genomic damage, thereby maintaining the plants’ phenotype. In line with previous report that low concentrations of EMS mutagenesis results in subtle phenotypic changes (Hu et al. 2017), but its application offers a high potential to increase the advantageous variation and generate alternative combinations of genotypes, which is of great importance for selective breeding of quinoa to adapt to different marginal environments.

Genome resequencing has been applied to investigate the mutations induced by mutagens in various crops, including SNPs and InDels (Shirasawa et al. 2016; Xiao et al. 2019; Tang et al. 2020). Typically, EMS induces G/C to A/T transition (Greene et al. 2003), and this type of variation was the most predominant among the nine mutants in this study, accounting for 34.4–67.2% of the total variation (Fig. 5). This observation consists with findings from other crops like *Triticum aestivum* (Wang et al. 2023), *Oryza sativa* (Yan et al. 2021), *Brassica rapa* ssp. *pekinensis* (Sun et al. 2022), and *Brassica napus* (Tang et al. 2020). Moreover, the SNPs induced by EMS mutagenesis are predominately located in intergenic regions, with 38.39% in *B. napus* (Tang et al. 2020), 53.9% in *B. rapa* ssp. *pekinensis* (Sun et al. 2022), and 22% in *Sorghum bicolor* (Jiao et al. 2016) being identified as such. In this study, 69.2–75.1% of SNPs were found in the intergenic regions (Fig. 5). It is important to note that sequence and codon preferences for EMS mutagenesis has been reported in *Oryza sativa* (Henry et al. 2014; Yan et al. 2021), indicating that similar preferences might also existed in quinoa.

### Electronic supplementary material

Below is the link to the electronic supplementary material.

Supplementary Material 1

## Data Availability

The datasets generated and/or analyzed during the current study are available in the NCBI with the BioSample number of SAMN36379215-SAMN36379223 for nine mutants and SAMN36379224-SAMN36379227 and SAMN31693738 for five Longli-4 wild type plants.
