# Supplementary material for: Genomic variation induced by a low concentration of ethyl methanesulfonate (EMS) in quinoa ‘Longli-4’ variety
Source: Bot Stud. 2024 Jul 5;65:15. doi: 10.1186/s40529-024-00427-x (PMC11226418; doi:10.1186/s40529-024-00427-x)
Supplement: Supplementary file 1 — Supplementary Material 1 [file 40529_2024_427_MOESM1_ESM.pdf]

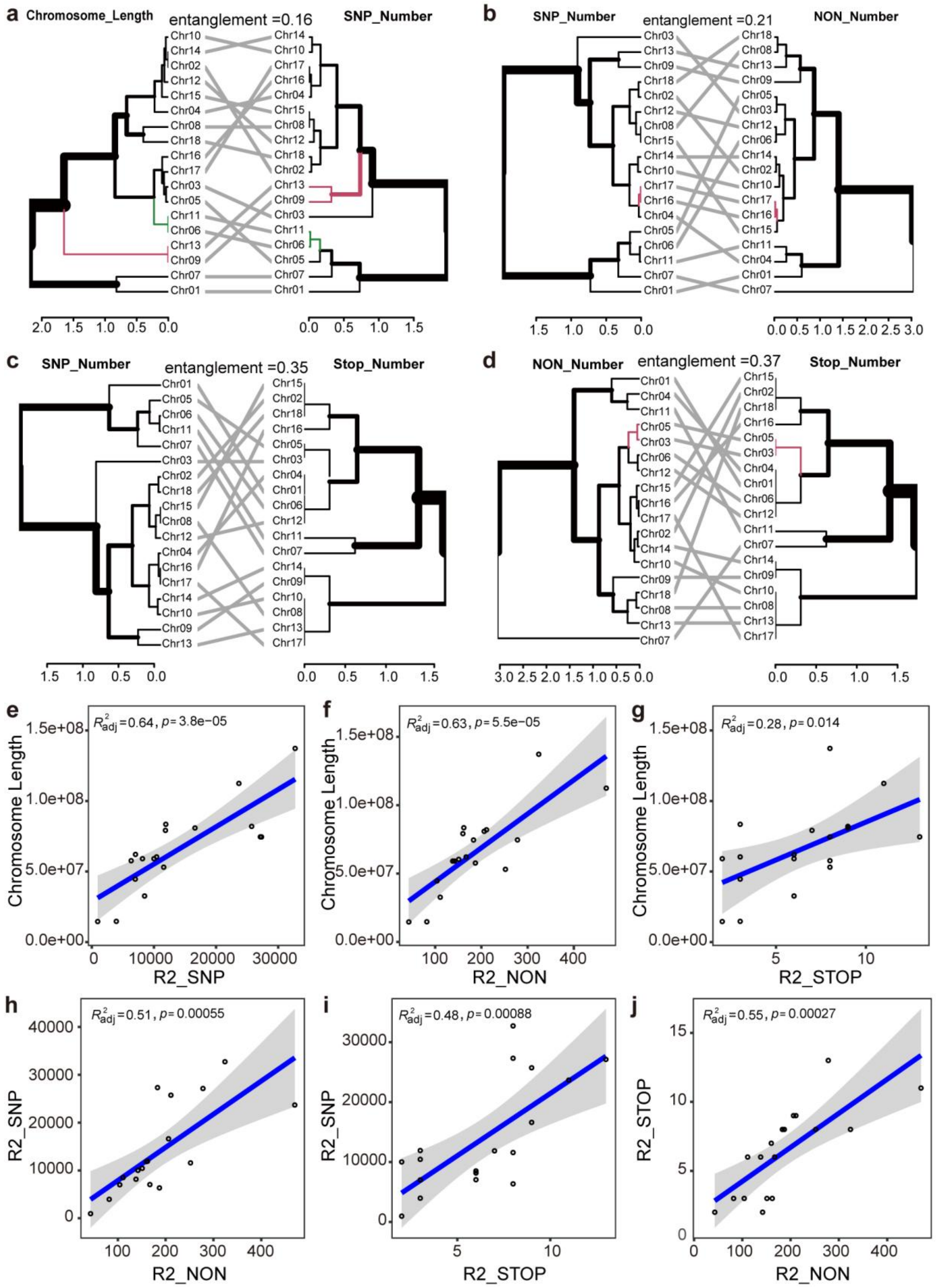

Fig. S1. Dendrogram and regression analyses of the chromosome length and SNPs number in R2. Dendrogram topology comparison of chromosome length and total SNPs number (**a**), and the pairwise comparisons of SNP, stop-gain, and non-synonymous mutation numbers (**b-d**); Linear regression diagram of chromosome length against total SNPs, non-synonymous, and stop-gain mutations (**e-f**), and the pairwise regression of SNPs, stop-gain, and non-synonymous mutation numbers (**h-j**). The

connection lines are in red and green (**a-d**) to highlight two sub-trees that are present in both dendrograms.

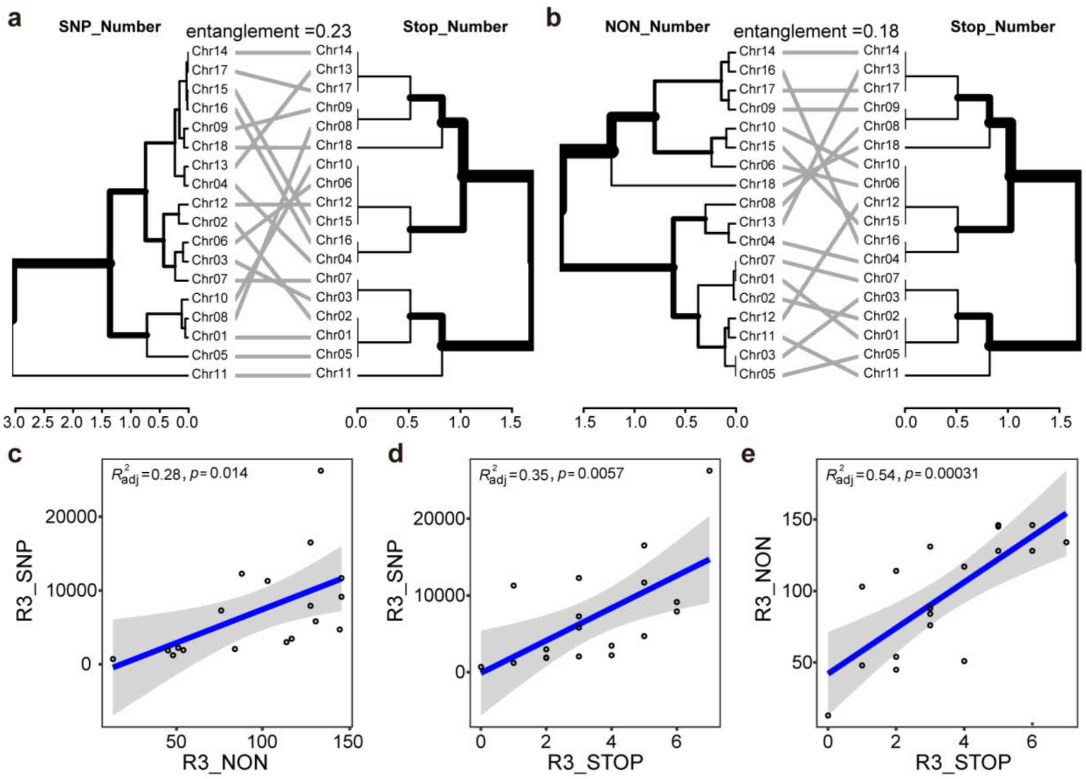

Fig. S2. Dendrogram and regression analyses of the chromosome length and SNPs number in R3. Dendrogram topology comparison between total SNPs and stop-gain number (**a**), and between non-synonymous mutation and stop-gain number (**b**); Linear regression diagram of the pairwise comparison of SNPs, stop-gain, and non-synonymous mutation number (**c-e**).

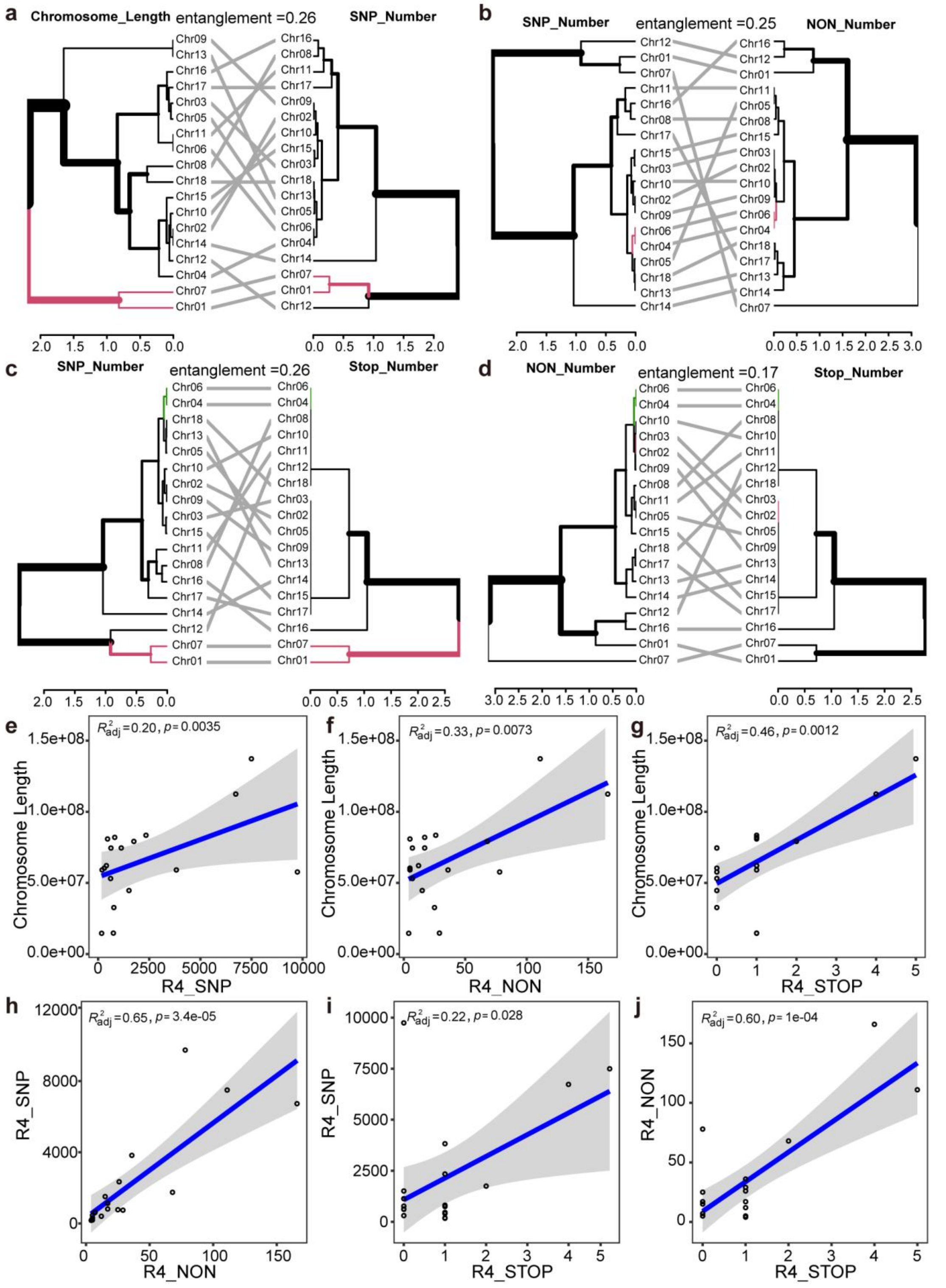

Fig. S3. Dendrogram and regression analyses of the chromosome length and SNPs number in R4. Dendrogram topology comparison of chromosome length and total SNPs number (**a**), and the pairwise comparisons of SNP, stop-gain, and non-synonymous mutation numbers (**b-d**); Linear regression diagram of chromosome length against total SNPs, non-synonymous, and stop-gain mutations (**e-f**), and the pairwise regression of SNPs, stop-gain, and non-synonymous mutation numbers (**h-j**). The

connection lines are in red and green (**a-d**) to highlight two sub-trees that are present in both dendrograms.

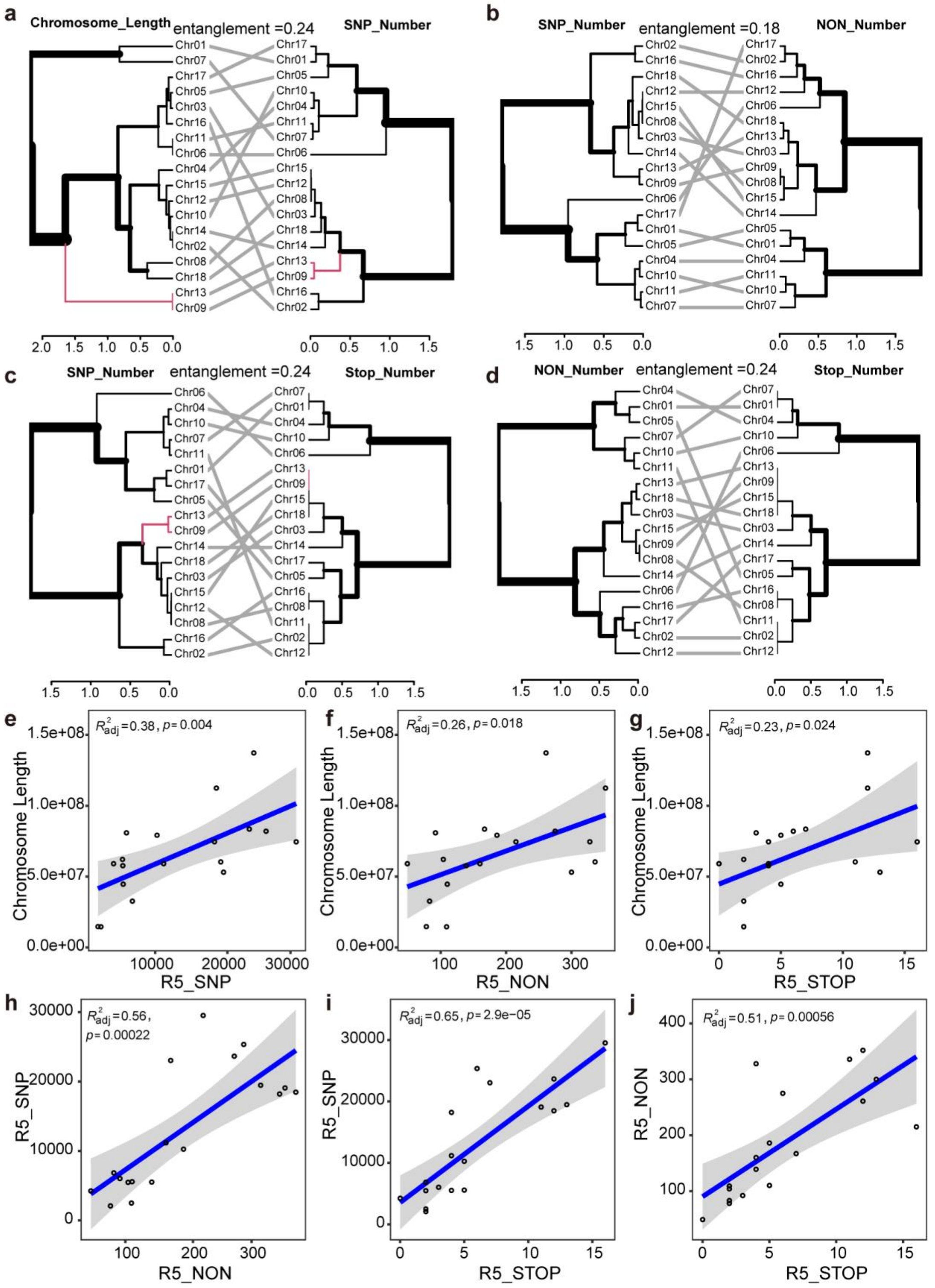

Fig. S4. Dendrogram and regression analyses of the chromosome length and SNPs number in R5. Dendrogram topology comparison of chromosome length and total SNPs number (**a**), and the pairwise

comparisons of SNP, stop-gain, and non-synonymous mutation numbers (**b-d**); Linear regression diagram of chromosome length against total SNPs, non-synonymous, and stop-gain mutations (**e-f**), and the pairwise regression of SNPs, stop-gain, and non-synonymous mutation numbers (**h-j**). The connection lines are in red (**a**, **c**) to highlight two sub-trees that are present in both dendrograms.

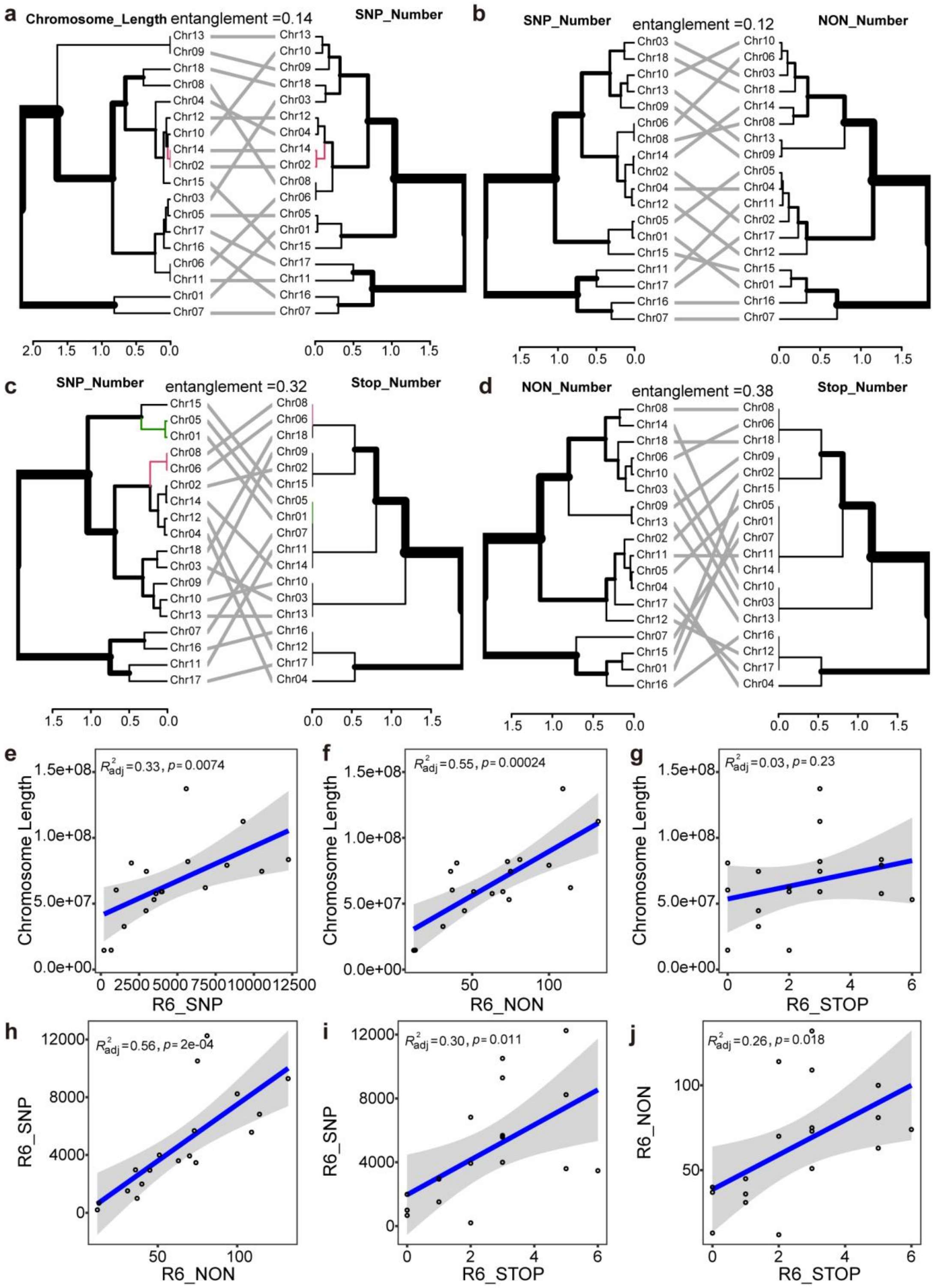

Fig. S5. Dendrogram and regression analyses of the chromosome length and SNPs number in R6. Dendrogram topology comparison of chromosome length and total SNPs number (**a**), and the pairwise comparisons of SNP, stop-gain, and non-synonymous mutation numbers (**b-d**); Linear regression diagram of chromosome length against total SNPs, non-synonymous, and stop-gain mutations (**e-f**), and the pairwise regression of SNPs, stop-gain, and non-synonymous mutation numbers (**h-j**). The connection lines are in red and green (**a**, **c**) to highlight two sub-trees that are present in both dendrograms.

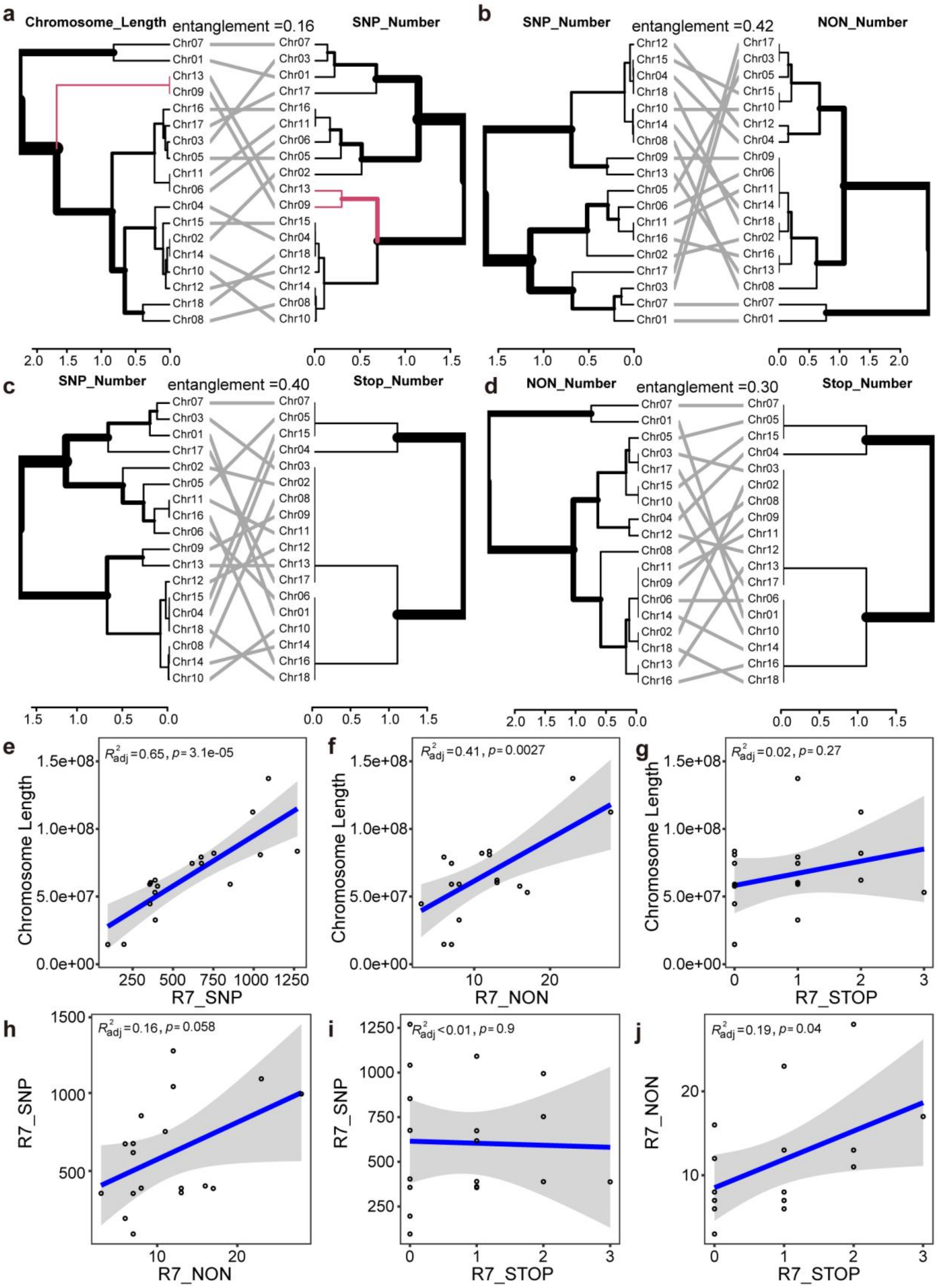

Fig. S6. Dendrogram and regression analyses of the chromosome length and SNPs number in R7. Dendrogram topology comparison of chromosome length and total SNPs number (**a**), and the pairwise comparisons of SNP, stop-gain, and non-synonymous mutation numbers (**b-d**); Linear regression diagram of chromosome length against total SNPs, non-synonymous, and stop-gain mutations (**e-f**), and the pairwise regression of SNPs, stop-gain, and non-synonymous mutation numbers (**h-j**). The

connection lines are in red (**a**) to highlight two sub-trees that are present in both dendrograms.

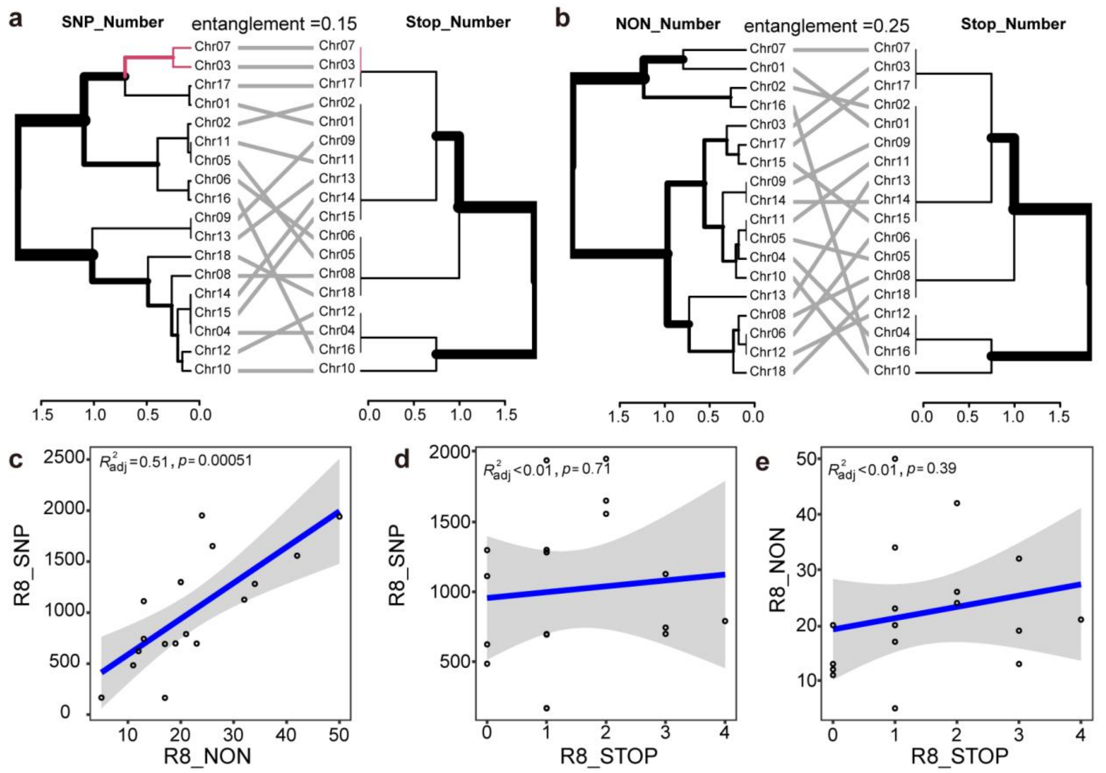

Fig. S7. Dendrogram and regression analyses of the chromosome length and SNPs number in R8. Dendrogram topology comparison between total SNPs and stop-gain number (**a**), and between non-synonymous mutation and stop-gain number (**b**); Linear regression diagram of the pairwise comparison of SNPs, stop-gain, and non-synonymous mutation number (**c-e**). The connection lines are in red (**a**) to highlight two sub-trees that are present in both dendrograms.

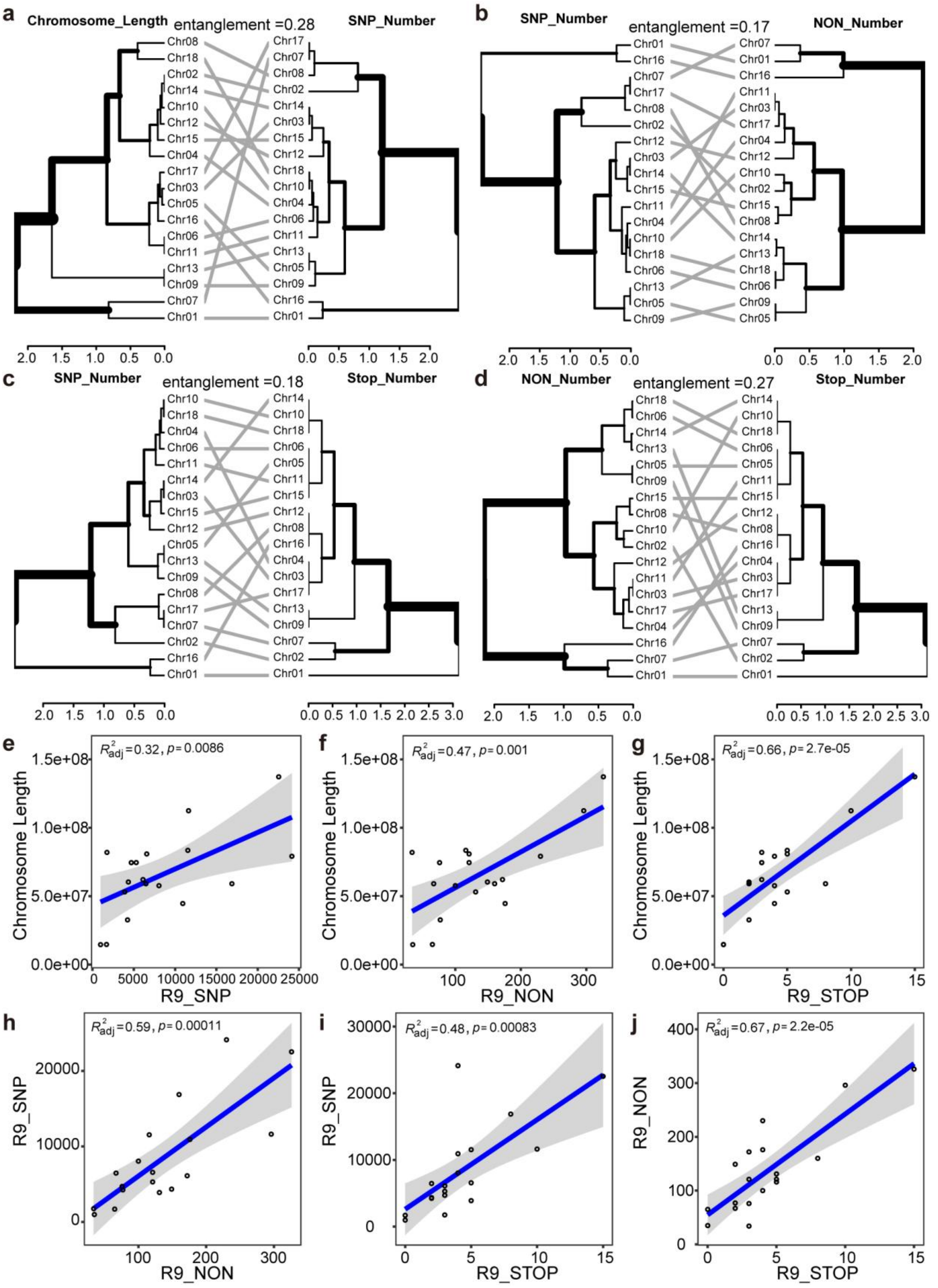

Fig. S8. Dendrogram and regression analyses of the chromosome length and SNPs number in R9. Dendrogram topology comparison of chromosome length and total SNPs number (**a**), and the pairwise comparisons of SNP, stop-gain, and non-synonymous mutation numbers (**b-d**); Linear regression diagram of chromosome length against total SNPs, non-synonymous, and stop-gain mutations (**e-f**), and the pairwise regression of SNPs, stop-gain, and non-synonymous mutation numbers (**h-j**).

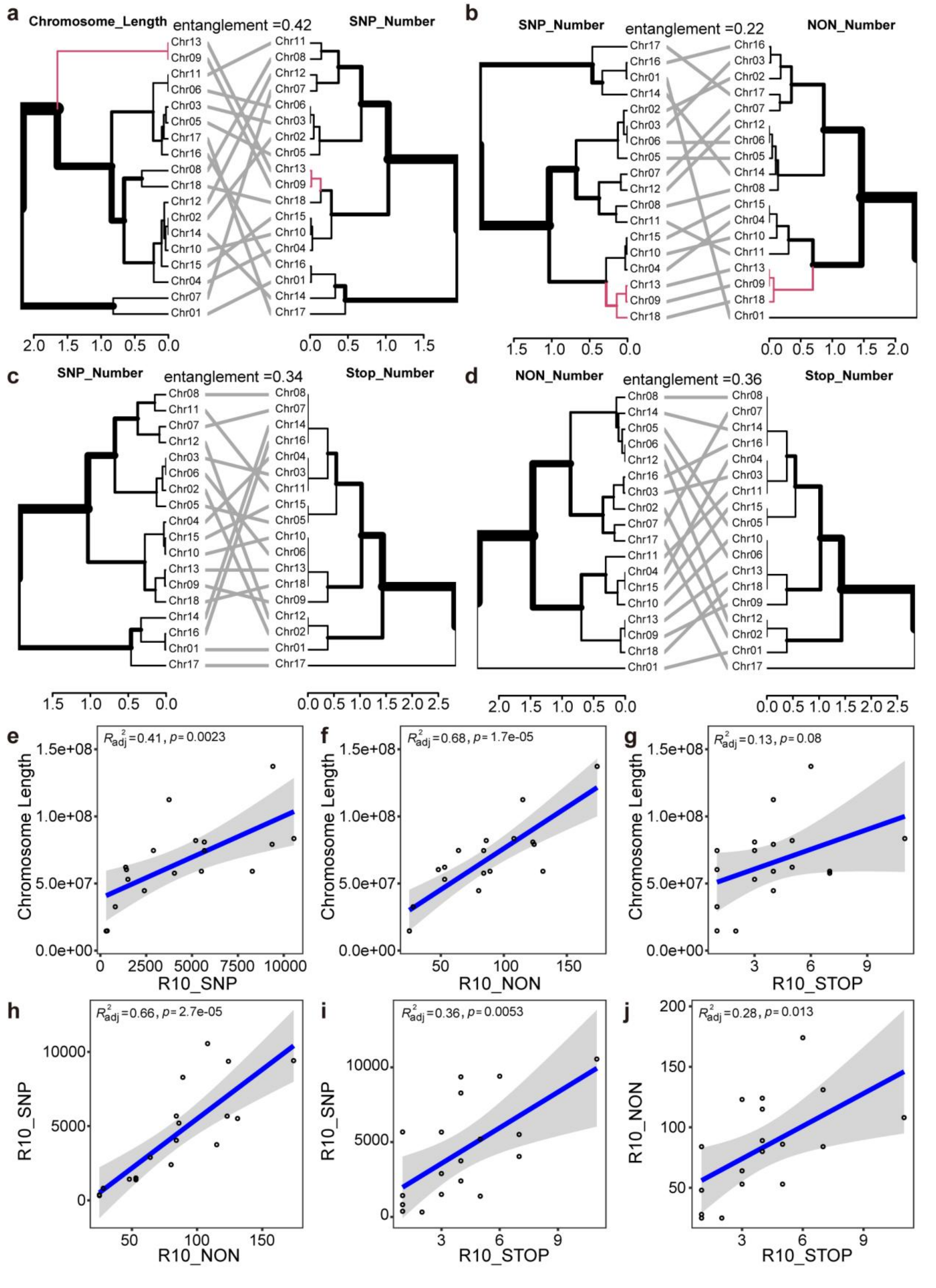

Fig. S9. Dendrogram and regression analyses of the chromosome length and SNPs number in R10. Dendrogram topology comparison of chromosome length and total SNPs number (**a**), and the pairwise comparisons of SNP, stop-gain, and non-synonymous mutation numbers (**b-d**); Linear regression diagram of chromosome length against total SNPs, non-synonymous, and stop-gain mutations (**e-f**), and the pairwise regression of SNPs, stop-gain, and non-synonymous mutation numbers (**h-j**). The

connection lines are in red (**a, b**) to highlight two sub-trees that are present in both dendrograms.

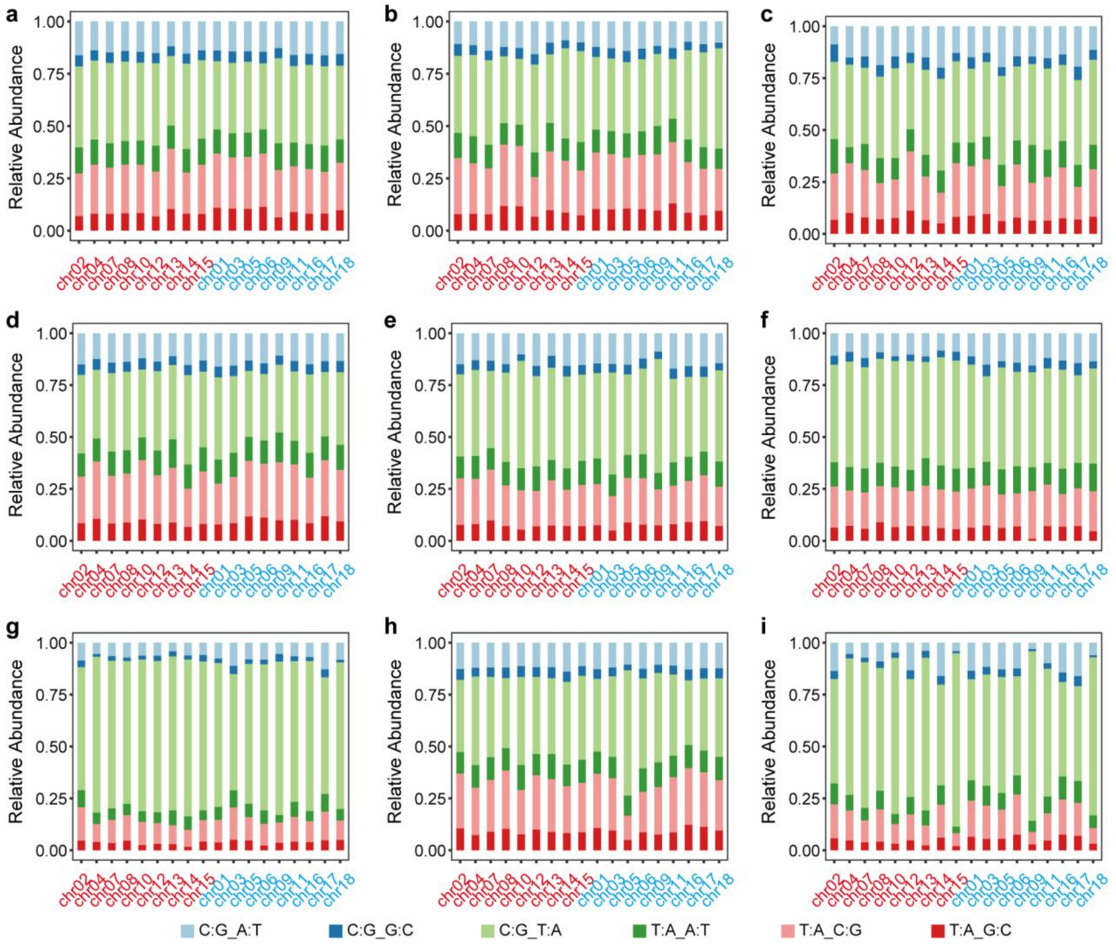

Fig. S10. SNPs identified individually in R2-R10 mutants against the Longli-4. (**a-i**) SNPs are categorized into six groups based on transitions and transversions, and the percentage of each group on each chromosome in R2-R10 mutants is presented as a stacked bar. The chromosomes of the A sub-genome are shown in red, and those of the B sub-genome are shown in pale blue.

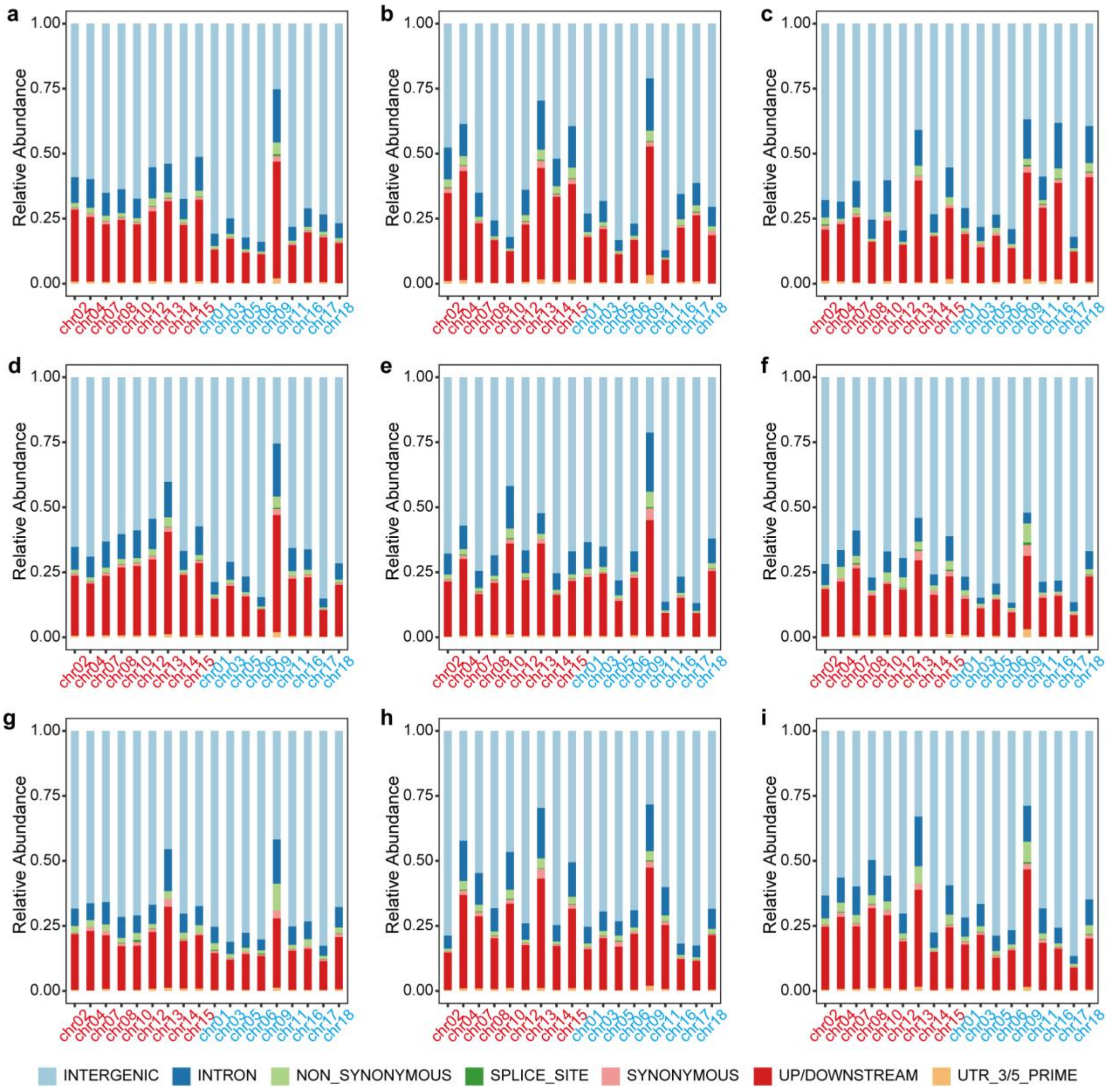

Fig. S11. Stacked bar charts of total variations in R2-R10 individuals. (**a-i**) SNPs are categorized into seven groups based on annotation, and relative abundance values of seven groups on each chromosome in R2-R10 are shown as a stacked bar. The chromosomes of the A sub-genome are shown in red, and those of the B sub-genome are shown in pale blue.
